# Beta oscillations in the sensorimotor cortex correlate with disease and remission in benign epilepsy with centrotemporal spikes

**DOI:** 10.1002/brb3.1237

**Published:** 2019-02-20

**Authors:** Dan Y. Song, Sally M. Stoyell, Erin E. Ross, Lauren M. Ostrowski, Emily L. Thorn, Steven M. Stufflebeam, Amy K. Morgan, Britt C. Emerton, Mark A. Kramer, Catherine J. Chu

**Affiliations:** ^1^ Department of Neurology Massachusetts General Hospital Boston Massachusetts; ^2^ Department of Radiology Massachusetts General Hospital Boston Massachusetts; ^3^ Athinoula A. Martinos Center for Biomedical Imaging Boston Massachusetts; ^4^ Harvard Medical School Boston Massachusetts; ^5^ Psychological Assessment Center Massachusetts General Hospital Boston Massachusetts; ^6^ Department of Mathematics and Statistics Boston University Boston Massachusetts

**Keywords:** BECTS, beta rhythms, biomarker, electrical source imaging, high density EEG, rolandic epilepsy

## Abstract

**Introduction:**

Benign epilepsy with centrotemporal spikes (BECTS) is a common form of childhood epilepsy with the majority of those afflicted remitting during their early teenage years. Seizures arise from the lower half of the sensorimotor cortex of the brain (e.g. seizure onset zone) and the abnormal epileptiform discharges observed increase during NREM sleep. To date no clinical factors reliably predict disease course, making determination of ongoing seizure risk a significant challenge. Prior work in BECTS have shown abnormalities in beta band (14.9–30 Hz) oscillations during movement and rest. Oscillations in this frequency band are modulated by state of consciousness and thought to reflect intrinsic inhibitory mechanisms.

**Methods:**

We used high density EEG and source localization techniques to examine beta band activity in the seizure onset zone (sensorimotor cortex) in a prospective cohort of children with BECTS and healthy controls during sleep. We hypothesized that beta power in the sensorimotor cortex would be different between patients and healthy controls, and that beta abnormalities would improve with resolution of disease in this self‐limited epilepsy syndrome. We further explored the specificity of our findings and correlation with clinical features. Statistical testing was performed using logistic and standard linear regression models.

**Results:**

We found that beta band power in the seizure onset zone is different between healthy controls and BECTS patients. We also found that a longer duration of time spent seizure‐free (corresponding to disease remission) correlates with lower beta power in the seizure onset zone. Exploratory spatial analysis suggests this effect is not restricted to the sensorimotor cortex. Exploratory frequency analysis suggests that this phenomenon is also observed in alpha and gamma range activity. We found no relationship between beta power and the presence or rate of epileptiform discharges in the sensorimotor cortex or a test of sensorimotor performance.

**Conclusion:**

These results provide evidence that cortical beta power in the seizure onset zone may provide a dynamic physiological biomarker of disease in BECTS.

## 1 INTRODUCTION

Benign epilepsy with centrotemporal spikes (BECTS) is a common childhood focal epilepsy syndrome characterized by a transient period of seizure susceptibility followed by sustained remission. The diagnosis of BECTS is based on stereotyped electroencephalogram (EEG) and clinical criteria (Scheffer et al., 2017). On EEG, children are found to have sleep‐activated epileptiform discharges in the central electrodes, corresponding to the sensorimotor cortex (Boor et al., 2007; Shiraishi et al., 2014). Clinically, seizures are usually brief, lasting for 1–3 min, typically occur during sleep, and manifest as somatosensory and motor symptoms mainly in the orofacial region with speech arrest and hypersalivation (Panayiotopoulos, Michael, Sanders, Valeta, & Koutroumanidis, 2008). Although BECTS is a common and well‐characterized epilepsy syndrome, seizure course, and disease duration are highly variable between children and there are currently no measures available to predict remission. Age of onset can range from 3–16 years, and remission typically occurs within 2–4 years and before the age of 16 years (Panayiotopoulos et al., 2008). While 15% of children will have only a single seizure, 85% may have recurrent seizures over several years (Bouma, Bovenkerk, Westendorp, & Brouwer, 1997). The perirolandic epileptiform spikes characteristic of this disease have been found to be unreliable indicators of seizure risk or remission (Kobayashi *et al*. 2010; Xie et al., 2018; Kim et al., 2018). Current clinical practice requires a trial‐and‐error method for administering anti‐epileptic drugs with wide variability and controversy over treatment strategy (Shields & Carter Snead III, 2009). Insufficient treatment can result in seizures, and rarely death due to sudden unexplained death in epilepsy (Doumlele et al., 2017). Conversely, unnecessary exposure to anticonvulsant drugs may introduce cognitive and physiological side effects during critical years of psychosocial and cognitive development, including attentional deficits, aggression, hostility, nervousness, and somnolence in exposed children (Halma et al., 2014; Masur et al., 2013; Perry, Holt, & Benatar, 2008). Given the uncertainty of disease course in BECTS, a physiological biomarker is needed to help identify risk of seizure recurrence or long term remission in these children.

Seizures in BECTS present during a time of maturational changes in cortical physiology which can be measured from noninvasive EEG studies. Prior work evaluating cortical rhythms in BECTS has found abnormalities in beta band power in the sensorimotor cortex during a motor task (Brindley et al., 2016) and rest (Koelewijn et al., 2015). Beta power is known to change heterochronically over childhood and thought to reflect normal cortical maturation (Chu, Leahy, Pathmanathan, Kramer, & Cash, 2014). Changes in beta power can also indicate state of consciousness (Engel & Fries, 2010) and can be modulated by GABA levels, which may be aberrant in seizure disorders (Baumgarten et al., 2016; Jensen et al., 2005; Khazipov et al., 2004). How and whether beta power relates to state of consciousness and seizure course has not been previously examined in BECTS.

Here, we evaluated whether cortical beta power may provide a dynamic physiological biomarker of disease in BECTS. We used high density EEG and source localization techniques to examine beta band activity in the seizure onset zone (sensorimotor cortex) in a prospective cohort of children with BECTS and healthy controls during sleep. We hypothesized that beta power in the sensorimotor cortex would be different between patients and healthy controls, and that beta abnormalities would improve with resolution of disease in this self‐limited epilepsy syndrome.

## 2 METHODS AND MATERIALS

### 2.1 Subjects

Twenty‐two children with BECTS (16 M, ages 7.2–14.9) and 11 school‐age healthy controls (HC, 3 M, ages 7.2–14.2) were recruited for this study. Patients were required to have a clinical diagnosis of BECTS by a board‐certified child neurologist following 1989 ILAE criteria (Commission on Classification & Terminology of the International League Against Epilepsy, 1989), a history of at least two clinical seizures characterized by focal facial motor activity or secondary generalized tonic clonic activity, and an EEG that showed sleep activated centrotemporal spikes. Children with attentional disorders and mild learning difficulties were included as these findings are consistent with the known cognitive deficits in BECTS (Wickens, Bowden, & D'Souza, 2017). Medication history, neurodevelopmental comorbidities, current medication status, and the month of the most recent seizure were recorded at the time of the EEG visit. Healthy control subjects were required to have no known history of epilepsy, neurological, genetic or psychiatric diseases, or intellectual disability. Subjects with a history of abnormal findings on neuroimaging were not eligible for inclusion in either group. Among BECTS subjects, the average number of years since first seizure to the EEG was 2.98 (range 0.1–9.06) and the average (range) number of years since last seizure was 1.35 year (range, 0–4.25 year). Clinical information on all subjects is listed in Table 1. Subjects and their guardians gave age‐appropriate informed consent according to standards reviewed by the Institutional Review Board at Massachusetts General Hospital.

**Table 1:** Subject and EEG data characteristics

Patient	Group	Age	Gender	Medication	Neurodevelopmental comorbidities	Duration from first seizure (years)	Duration seizure‐free (years)	Wake EEG length (s)	Sleep EEG length (s)	Centrotemporal spikes (Y/N)/lateralization (L/R/B)
1	BECTS	13.7	F	None	ADHD	7.03	4.25	200	200	Y/B
2	BECTS	11.8	M	LEV	None	1.67	1.42	200	200	N
3	BECTS	14.7	M	None	Learning disorder	4.34	0	200	200	Y/L
4	BECTS	14.9	M	None	ADHD	9.06	3.17	200	200	Y/L
5	BECTS	13.3	M	LEV	None	4.27	2.17	187	200	Y/R
6	BECTS	9.1	F	LEV, LTG	ADHD, Learning disorder, Auditory processing disorder	0.89	0.08	200	200	Y/B
7	BECTS	9.8	M	OXC	None	0.10	0	134	200	Y/R
8	BECTS	12.8	F	None	ADD	4.97	2.83	200	200	N
9	BECTS	8.0	M	None	None	2.64	2.42	200	200	Y/B
10	BECTS	14.8	M	None	None	6.89	3.33	200	N/A	N
11	BECTS	11.0	F	None	None	2.31	0.17	200	199	Y/B
12	BECTS	9.0	M	None	None	0.71	0.33	131	105	Y/B
13	BECTS	10.9	M	None	None	2.58	0.83	200	185	Y/B
14	BECTS	11.5	M	None	None	1.81	1.67	N/A	200	Y/L
15	BECTS	11.6	M	LEV	None	0.72	0.17	200	113	N
16	BECTS	10.5	F	LEV	Learning Disorder	4.07	0.33	188	103	Y/R
17	BECTS	10.4	M	LEV	Language Disorder	2.48	2.17	198	161	Y/B
18	BECTS	11.9	M	LEV	None	4.53	2	200	200	N
19	BECTS	11.6	M	None	None	1.15	1.17	200	200	Y/R
20	BECTS	9.9	M	None	None	2.29	0.5	200	200	Y/B
21	BECTS	11.3	M	None	ADHD, Learning disorder	0.48	0.08	200	200	Y/B
22	BECTS	9.6	M	None	Dyslexia, ADHD	0.61	0.58	134	200	Y/B
1	HC	9.0	F	N/A	None			200	200	
2	HC	7.2	F	N/A	None			200	N/A	
3	HC	7.9	M	N/A	None			200	N/A	
4	HC	8.3	M	N/A	ADHD			200	N/A	
5	HC	12.9	F	N/A	None			200	200	
6	HC	12.2	F	N/A	None			192	200	
7	HC	14.2	F	N/A	None			200	200	
8	HC	9.4	F	N/A	None			200	200	
9	HC	9.4	F	N/A	None			127	35 s	
10	HC	13.6	F	N/A	Learning disorder			189	200	
11	HC	9.4	M	N/A	None			128	200	

LEV: levetiracetam; LTG: lamotrigine; OXC: oxcarbazepine; ADHD: attention deficit hyperactivity disorder; Y: yes; N: No; L: left; R: right; B: bilateral.

### 2.2 EEG data collection and preprocessing

All subjects were instructed to follow a sleep‐deprivation protocol prior to their recording session, with a recommendation to restrict sleep to 4 hr the night prior. Resting state EEG data were collected with a 70‐channel electrode cap, at a sampling rate of 2,035 Hz. Prior to recording, EEG electrode positions were digitized using a 3‐D digitizer (Fastrak, Polhemus Inc., Colchester, VA). Subjects were then recorded in a quiet, resting state with eyes closed until 10 min of NREM sleep was obtained or 2 hr passed, whichever came first.

**Figure 1:**
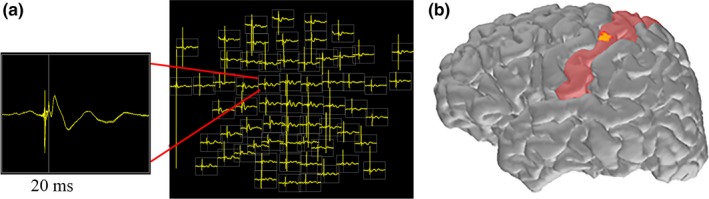
(a) Example EEG field map of averaged activity from ~100 right median nerve stimulations in a single subject. The N20 peak is confirmed through visual analysis. (b) Electrical source localization is performed utilizing the patients MRI data and co‐registered electrode coordinates. Visual inspection of the N20 peak (yellow) confirms localization to the postcentral gyrus (primary sensory cortex) hand representation area

After the resting EEG session was completed, somatosensory evoked potentials (SEP) from the median nerve from each arm were recorded to localize the sensorimotor cortex and confirm EEG‐MRI co‐registration accuracy (Forss et al., 1994; Yao & Dewald, 2005). Stimulation voltages were increased until the motor threshold was reached (2.5–3.5 V). If the motor threshold was not reached due to subject discomfort, subjects confirmed stimulus sensations were present in the thumb to ensure the median nerve was stimulated. Approximately 100 stimulations over 4 min were delivered to the left and right median nerves with a random interstimulus interval between 1,400 and 1600 ms. If insufficient time was available or if subjects did not tolerate SEP recording, they were omitted from the recording session. As small changes in EEG sensor location due to subject movement may impact EEG source space estimates, for all subjects with SEPs that showed clear n20 and p35 peaks (*n* = 18 BECTS, *n* = 4 HCs), we localized each subject's recorded SEPs and confirmed accurate localization to the post central gyrus using MNE software (Gramfort et al., 2014). An example of SSEP source localization for one patient using this procedure is shown in Figure 1.

EEG data were manually reviewed by a board‐certified clinical neurophysiologist to identify epochs of wake and sleep according to standard criteria (Silber et al., 2007) and to manually mark interictal spikes. Most BECTS subjects (17/22) had centrotemporal spikes on their study sleep EEG recording; of these 10 had bilateral independent spikes, 4 had right centrotemporal spikes, and 3 had left centrotemporal spikes (Table 1). All available NREM sleep epochs were selected for subsequent analysis. Subjects that did not have NREM data were excluded from sleep state analyses. Spectrograms (1 s windows) of the EEG data for all electrodes were then visually inspected to remove all 1 s epochs contaminated by movement, muscle, and electrode artifacts, which appear as visually apparent anomalous activity or spectral streaks that do not follow expected 1/f properties of brain oscillations (Chu et al., 2014; Freeman, Rogers, Holmes, & Silbergeld, 2000; Gasser, Schuller, & Gasser, 2005). As sharp events are known to impact the estimate of power at all frequencies, especially at high frequencies (He, Zempel, Snyder, & Raichle, 2010; Kramer, Tort, & Kopell, 2008), all 1 s time intervals that overlapped with ±50 ms around interictal spike peaks were ignored. Channels containing any artifacts or with poor recording quality for the entire recording were removed from analysis. A minimum of 100 s of artifact free data were used for analyses (wake: mean 189 s, range 131–200 s; sleep: mean 184 s, range 105–200 s). This minimum epoch duration has been demonstrated to be sufficient to identify stable EEG physiological brain signals (Chu et al., 2012).

### 2.3 MRI data collection and preprocessing

Among all 33 subjects, two subjects could not tolerate the MRI scan and one subject's MRI was not usable due to gross motion artifact affecting surface reconstruction, leaving a total of 30 subjects with MRI data. MRI data were collected on the same day as the EEG data for 22 subjects; eight subjects did not have same day recording due to subject and scanner schedules and MRI data were recorded in a subsequent visit (mean duration to next visit: 3.8 days, range: 0–36 days). T1‐weighted multi‐echo magnetization‐prepared rapid acquisition gradient‐echo (MEMPRAGE) images were collected on a 3 T MAGNETOM Prisma Scanner (Siemens, Germany) with the following parameters: TR = 2,530 ms, TE = (1.69, 3.55, 5.41, 7.27 ms), voxel size 1x1x1 mm, flip angle =7 degrees. Minor distortions in the original volume image due to nonlinearities in the MRI gradient specific to the hardware used in our scanner were corrected prior to analysis using interpolation in a custom MATLAB script.

### 2.4 Sensorimotor performance testing

A Grooved Pegboard task administered by a board‐certified (AKM) or board‐eligible (BCE) neuropsychologist as close in time to the EEG recording as feasible (mean 28.6 days, range 0–142 days). This task provides a quantitative evaluation of motor speed during complex sensorimotor function in the dominant hand.

### 2.5 Source space beta power calculation

In order to improve the spatial resolution of our analysis, we estimated the brain electrical activity on the cortical surface and computed beta power from anatomically designated regions of interest (ROIs) in each subject. Source analysis of EEG data was performed using the MNE software package (Gramfort et al., 2014; Hamalainen & Sarvas, 1989; Sharon, Hämäläinen, Tootell, Halgren, & Belliveau, 2007) with anatomical surfaces reconstructed using Freesurfer (Fischl, 2012) following previously described methods (Chu et al., 2015). Briefly, for the forward model, a three‐layer boundary element model (BEM) consisting of the inner skull, outer skull, and outer skin surfaces with electrical conductivities of 0.33 S/m, 0.006 S/m, and 0.33 S/m, respectively, was generated (Hamalainen & Sarvas, 1989). The digitized EEG electrode coordinates were co‐registered to the reconstructed surface using the nasion and auricular points as fiducial markers. To generate the solution space, a subdivided icosahedron was fitted to the cortical surface inflated to the shape of a sphere. This generated a three‐dimensional grid with 4,098 vertices per hemisphere (8,196 total) from which sources activity was inferred. The inverse operator was computed from the forward solution with a loose orientation constraint of 0.6 to eliminate implausible sources and 2 microvolts as the estimate of EEG noise.

All artifact‐free 1 s EEG time intervals were used in the source space analysis. For each ROI, after source estimates for the entire cortical surface were inferred, all cortical sources within the ROI were averaged, and the mean beta power per ROI computed. The power spectrum from data recorded during wake and sleep states were calculated with nonoverlapping 1 s windows using the MATLAB function *fft* and a Hanning taper, giving ~1 Hz frequency resolution.

**Figure 2:**
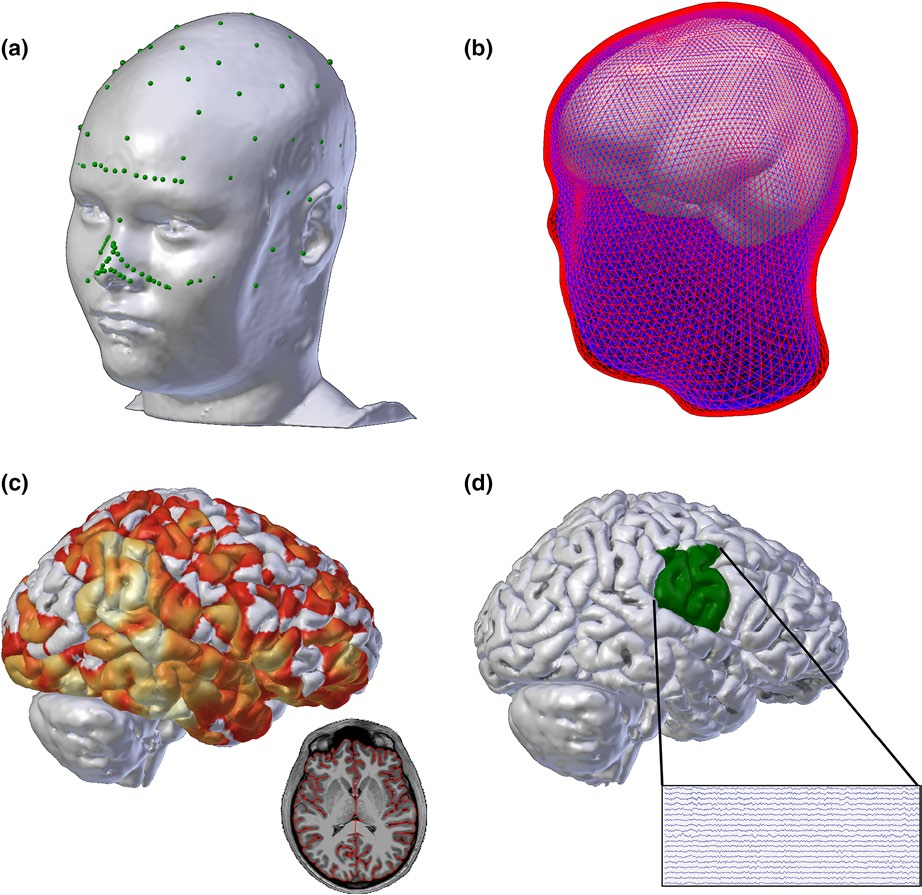
(a) EEG electrode coordinates (green) are co‐registered to a detailed head model generated by each subject's MRI. (b) Boundary element models of the outer skin (red), outer skull (blue), and inner skull (white) layers are generated for the forward solution model. (c) Example sourcedata at one time point; the solution is constrained to the cortex as shown in the axial MRI slice inset (colors indicate current magnitude). (d) Source data in the seizure onset zone (e.g. the lower half of the sensorimotor cortex) are selected for analysis (example cortical source activity time series recording shown in inset)

Beta band power in each ROI in the right and left hemispheres were averaged together to achieve a single measure of beta power for each subject. The power of the source data was calculated in picoamps and then log scaled (Kramer & Eden, 2016). The resulting power had units of log10(pA^2^/Hz). Figure 2 outlines the process for inference and calculation of source space beta activity which was visualized using the Multi‐Modality Visualization Tool (LaPlante RA et al., 2017).

### 2.6 Selection of ROIs

Our primary ROIs were the lower half of the postcentral and precentral gyri, which approximate the seizure onset zone. Although there may be subtle individual variations, BECTS is a unique idiopathic focal developmental epilepsy characterized by stereotyped seizure semiology and spike features in which the epileptogenic region has been found to consistently localize to the lower half of the perirolandic cortex using EEG, MEG, and fMRI source localization techniques (Boor et al., 2007; Pataraia, Feucht, Lindinger, Aull‐Watschinger, & Baumgartner, 2008). As beta activity is variable across brain regions (for example see Chu et al., 2015), we chose this anatomically consistent ROI across subjects to allow for across subject comparison between groups.

**Figure 3:**
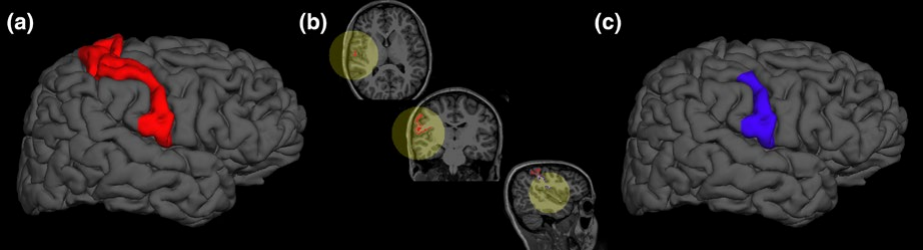
To isolate the lower half of the peri‐rolandic cortex, after registering the MRIs to a shared coordinate system, all cortical regions shared by pre and postcentral gyri (red, a) and a sphere centered on the most inferior vertex in the rolandic cortex and a radius equal to half of the distance between the most superior and inferior vertices in the rolandic cortex (yellow, b), were included in the final seizure onset zone label (blue, c)

To generate labels for these regions, vertices in the postcentral gyrus and precentral gyrus were labeled via the Desikan‐Killiany Atlas (Desikan et al., 2006). A Right‐Anterior‐Superior (RAS) coordinate system centered at the anterior commissure was generated using Freesurfer. Using custom MATLAB software, the distance between the most superior and inferior vertices in the pre and postcentral gyri labels along the coronal plane was calculated. We then created a separate label using a sphere with the center focused at the most inferior vertex and the radius equal to half of the distance between the most superior and inferior points in the rolandic cortex. All vertices shared by the sphere label and the pre and postcentral gyri labels were included in the custom labels evaluating the lower half of the pre and postcentral gyri. The steps to generate this custom label are outlined in Figure 3. Resulting ROIs were visually inspected to confirm accuracy. All other labels used in exploratory analysis were generated directly from the Desikan‐Killiany Altas (Desikan et al., 2006).

### 2.7 Sensor space beta power calculation

After source space analysis, we also explored whether our findings were evident in EEG sensor space, which is the most common signal evaluated in the clinical setting. For this analysis, to improve the focality of the signal analyzed and minimize the impact of volume conduction, skull thickness, and other noncortical contributions to the signal, data were re‐referenced to the bipolar montage (Lepage, Kramer, & Chu, 2014; Nunez & Srinivasan, 2006). The power spectrum from data recorded during wake and sleep states were calculated following the procedures used for the source space time series analysis.

In this focal epilepsy syndrome, epileptiform spikes arise independently in the left and right hemisphere, and localize to the rolandic cortex (Lin et al., 2003). Thus, we focused our analysis on bipolar recordings from adjacent electrodes in the sensorimotor cortical regions of the left and right hemispheres, C3‐C5 and C4‐C6, respectively. The average power values in the beta band (14.9–30 Hz) at each bipolar channel pair were computed in microvolts for every 1 s time interval in each available arousal state. These values were averaged across time intervals and hemispheres and log scaled to achieve a single measure of beta band power per subject per state. If one of the four electrodes was removed during the preprocessing stage due to excessive artifacts, its bipolar pairing was excluded from analysis (e.g. if C3 contained artifacts, only the bipolar pair C4‐C6 was used).

### 2.8 Statistical analysis

To mitigate the impact of false positive results following from the multiple testing problem, we tested two a priori hypotheses: (a) that the source estimated beta power during sleep in the seizure onset zone is different in children with BECTS compared to healthy control children; and (b) that source estimated beta power during sleep in the seizure onset zone predicts duration seizure‐free in patients with BECTS.

Group comparisons were performed using a logistic regression model, with beta band power and age as the predictors and group as the dependent variable. To evaluate whether beta power correlates with duration seizure‐free among BECTS subjects, we performed standard linear regression with the identity distribution as the link function, with beta power as the predictor and duration seizure‐free as the dependent variable. For a priori tests, significance was set at *p* < 0.05.

Upon identifying a difference in beta source power, we explored the specificity of our findings and correlation with clinical features using logistic and standard linear regression models. As these tests were investigated post‐hoc, they are reported as exploratory results. For all a priori and post‐hoc tests with group or duration seizure‐free evaluated as the dependent variables, age was tested as a predictor and included in a multivariate model as a covariate if found to be significant. Among BECTS subjects, medication status was not a significant predictor of duration seizure‐free, and therefore was not included in the analysis. Adjusted and unadjusted *p*‐values are reported, when appropriate and also provided in Table 2 and Tables S1–S3.

**Table 2:** Univariate and multivariate tests to evaluate for a relationship between beta power and group and duration seizure‐free

	Dependent variable	Univariate beta power	Univariate age	Multivariate beta power + age
Logistic regression	Healthy control versus BECTS	*p* = 0.887 (wake)	*p* = 0.185	
		***p* = 0.030 (sleep)** a	*p* = 0.846	
Linear regression	Duration seizure‐free	*p* = 0.008 (wake)	*p* = 0.003	**Beta *p *= 0.014** b
		*p* = 0.011 (sleep)	*p* = 0.011	**Beta *p *= 0.027** a

Note

Unadjusted *p*‐values (column 2) and, when appropriate, adjusted *p*‐values accounting for age (column 3) are reported.

a Significant value in an a priori test.

b Possible relationship identified in exploratory testing

## 3 RESULTS

### 3.1 Source space beta power in the seizure onset zone is higher in BECTS children during sleep

**Figure 4:**
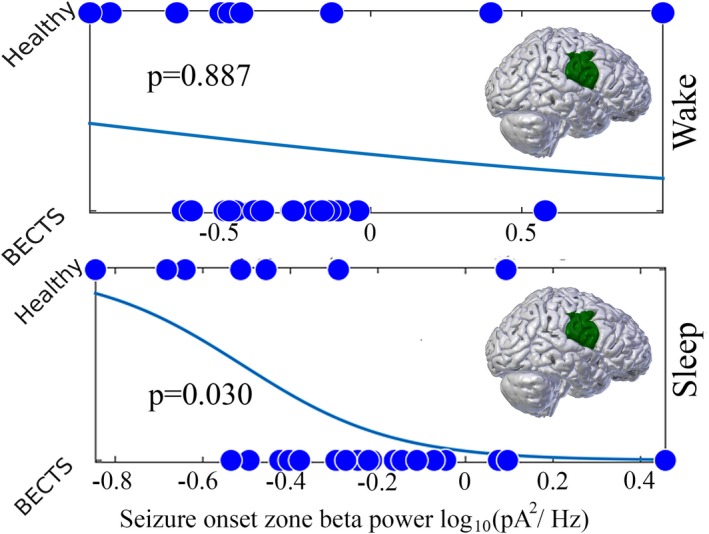
Logistic regression models of group (BECTS or healthy control subjects) using electrical source imaging estimates of beta power in the seizure onset zone. The beta power is a significant predictor of group during sleep (bottom) but not wake (top)

We evaluated for differences in beta power between BECTS and healthy controls in the seizure onset zone (lower half of the sensorimotor cortex). We found that beta power in the seizure onset zone predicted group, where BECTS (*n* = 19) had higher beta power compared to healthy controls (*n* = 7, *p* = 0.030, Figure 4, Table 2) during sleep.

### 3.2 Beta band power versus duration seizure‐free in the seizure onset zone

In BECTS, the majority of children who sustain at least 1 year seizure‐free will not relapse and have successfully entered disease remission (Berg et al., 2004). Those who are seizure‐free for 2 years are even less likely to have a seizure and more likely to have entered sustained remission (Berg et al., 2001, 2004). Thus, we used duration of time (months) seizure‐free as a continuous variable to reflect likelihood of remission, where the longer a child has been seizure‐free, the more likely that child has entered remission.

**Figure 5:**
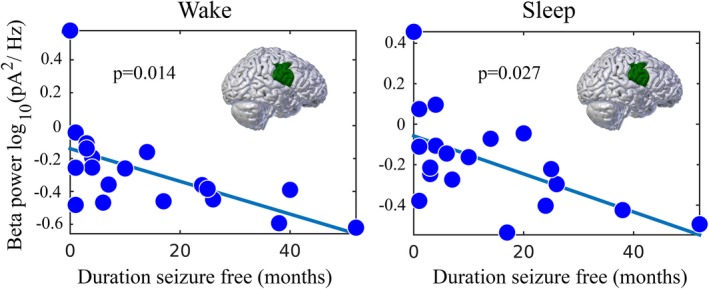
Linear regression models of electrical source imaging estimates of beta power in the seizure onset zone in BECTS, with predictor of duration seizure‐free. Beta power in the seizure onset zone is significantly correlated with duration seizure‐free

We found a significant relationship between beta power in the seizure onset zone and duration seizure‐free measured during sleep (*n* = 19, unadjusted *p* = 0.011; adjusted *p* = 0.027, Figure 5).

## 4 SUPPLEMENTAL ANALYSIS

### 4.1 State specificity

To explore the specificity of our findings to the sleep state, we investigated whether the relationships we observed between beta power and disease status (e.g. BECTS vs. healthy control) as well as duration seizure‐free were observed during wakefulness. We found that BECTS subjects (*n* = 19) did not have higher beta in the seizure onset zone compared to healthy controls (*n* = 10, *p* = 0.887, Figure 4) during wakefulness. Among BECTS subjects, we did find a possible relationship between beta power in the seizure onset zone and duration seizure‐free measured during wake (*n* = 19 *p* = 0.008 unadjusted, *p* = 0.014, adjusted, Table 2, Figure 5).

### 4.2 Spatial specificity

To explore the spatial specificity of our findings, we investigated whether the relationships we observed between beta power and disease status (e.g. BECTS vs. healthy control) as well as duration seizure‐free were unique to the seizure onset zone in the sensorimotor cortex (Figure S1, Table S1). For this analysis, we evaluated regional and global brain cortical beta power. For these estimates, we generated a solution space of 162 sources distributed per hemisphere (324 total). For a global brain beta power estimate, the beta power was first computed at each source space point for each 1 s interval, then these values were averaged in space and in time. For lobar beta power estimates, ROIs for the four major lobes (frontal, temporal, parietal, and occipital) were created using the Desikan‐Killiany atlas, and the beta power at each of the source space points were averaged within each lobar ROI and across all 1 s intervals. These values were computed in picoamps and then log scaled resulting in units of log10(pA^2^/Hz). We note that the precentral and postcentral gyri were not included in the frontal and parietal lobe ROIs.

Because only beta power measured during sleep was found to differ significantly between BECTS and HCs, we analyzed the spatial specificity of these findings only in sleep. We found that beta power in the temporal and parietal lobes was a possible predictor of group (*p* = 0.037, *p* = 0.028, respectively; Figure S1) and a possible trend of global beta power to predict group (*p* = 0.051). Beta power in the frontal and occipital lobes did not predict group status (*p* = 0.176 and 0.189, respectively).

We also found a possible relationship between global source space beta power and duration seizure‐free in both wakefulness (*p* = 0.043, unadjusted; *p* = 0.011, adjusted) and sleep (*p* = 0.026 unadjusted; *p* = 0.015, adjusted) in BECTS subjects (*n* = 19). In lobar analysis, this relationship was also apparent in both arousal states in the frontal lobe (wake *p* = 0.020, sleep *p* = 0.018). Beta power in the temporal, parietal, and occipital lobes, did not correlate with duration seizure‐free in either wakefulness or sleep (*p* > 0.07).

### 4.3 Frequency specificity

To evaluate whether the relationships between brain rhythms and epilepsy in BECTS were beta‐specific phenomena, we explored power in four additional conventional EEG frequency bands: delta (0.9–4 Hz), theta (3.9–8 Hz), alpha (7.9–12 Hz), and gamma (29.9–50 Hz; Figure S2, Table S2).

We assessed whether these frequency bands measured during sleep in the sensorimotor cortex in source space predicted group status (BECTS vs. HCs). Delta and gamma power during sleep were not significant predictors of groups status after correcting for age (*p* = 0.251, *p* = 0.101), while theta trended toward a possible relationship (*p* = 0.052) and alpha power was a possible predictor of group status during sleep (*p* = 0.024).

We also found a possible relationship between duration seizure‐free and power in the alpha band during wake (*p* = 0.010 unadjusted, *p* = 0.044 adjusted) and sleep (*p* = 0.002 unadjusted, *p* = 0.027 adjusted). Gamma power during sleep also showed a possible relationship with duration seizure‐free (*p* = 0.021 unadjusted; *p* = 0.013 adjusted).

### 4.4 Sensor space analysis

To explore whether our findings could be observed in sensor space data, we evaluated beta power in the central electrodes and bipolar channel subsets from each lobe (Figure S3, Table S3). We found no difference in sensor space central electrode beta power between healthy controls and BECTS subjects during wakefulness (HC = 11, BECTS = 21, *p* = 0.523) or sleep (HC = 8, BECTS = 21, *p* = 0.264). We observed no relationship between duration seizure‐free and beta power in sensor space data during wakefulness (*n* = 21, *p* = 0.040 unadjusted; *p* = 0.336 adjusted) or sleep (*n* = 21, *p* = 0.011, unadjusted; *p* = 0.061, adjusted).

Given the potential clinical utility of identifying a biomarker in sensor space alone, we explored power in delta, theta, alpha, and gamma frequencies at the central electrodes during sleep and found no relationship with duration seizure‐free (*p* > 0.08 for all adjusted tests). To evaluate for a diffuse abnormality in sensor space, we computed the average beta power across a sampling of bipolar channels (two channels from each lobe from each hemisphere: F3‐F5, F4‐F6, T3‐TP7, T4‐TP8, P3‐P5, P4‐P6, O1‐PO3, O2‐PO4) during wakefulness and sleep and also found no relationship with duration seizure‐free (unadjusted *p* = 0.339 and *p* = 0.07, respectively; adjusted, *p* = 0.96 and *p* = 0.20, respectively).

### 4.5 Relationship between beta power and clinical variables

We explored whether there was a relationship between beta power and several clinical variables (Table S4). Among all BECTS subjects, there was no relationship between source space beta power in sleep in the seizure onset zone and the presence of epileptiform spikes (*p* = 0.878). There was a trend toward a relationship between beta power and spike rate (*p* = 0.053), but this trend disappeared when age was included in the regression (*p* = 0.20).

Among all subjects, there was no relationship between whole brain source space beta power during sleep and the presence of a neuropsychological diagnosis (*p* = 0.674). There was no relationship between dominant hemisphere rolandic beta power and dominant hand grooved pegboard performance (*p* = 0.540).

## 5 DISCUSSION

Here we evaluated a cohort of children with a well‐characterized, focal epilepsy syndrome using sophisticated electrical source imaging techniques and demonstrate a relationship between resting cortical oscillations, disease state, and ongoing seizure risk. We found that abnormal beta frequency cortical rhythms were present during sleep in the seizure onset zone in children with BECTS compared to healthy control children. We further found that, among children with BECTS, beta power correlated with duration seizure‐free, thereby reflecting a dynamic biomarker that recovers in conjunction with disease remission.

The analysis of a BECTS population provides several advantages to our study design. Because children in this population exhibit disease progression from epilepsy to remission, this allows the evaluation of a biomarker to identify disease and dynamically track disease status. Furthermore, the consistent focality of the epileptogenic region amongst BECTS patients allows creation of consistent ROIs across the population. This consistency allows us to formulate our hypothesis a priori*,* which minimizes the risk of false positive findings. The lack of a spatially focal target is a particularly important limitation to studies assessing activity across broad brain regions using voxel wise comparisons or other exploratory approaches.

Because cortical rhythms are known to change dramatically over childhood (Chu et al., 2012), these findings may in part be driven by an atypical trajectory of inherent developmentally mediated changes in cortical excitability (Baho & Di Cristo, 2012; Le Magueresse & Monyer, 2013). Alternatively, the changes in cortical rhythms observed here could reflect abnormalities in global cortical volume (Kim et al., 2015; Pardoe, Berg, Archer, Fulbright, & Jackson, 2013), cortical thickness (Overvliet et al., 2013), or white matter microstructure or connectivity (Ciumas et al., 2014; Kim, Lee, Chung, Lim, & Lee, 2014; Xiao et al., 2014) that have been reported in children with BECTS. Irrespective of mechanism, we find that cortical power estimates extracted from EEG, an easily acquirable noninvasive modality, track with disease and disease course, where reduced activity in the beta frequency range during sleep could indicate that a patient is entering disease remission. Furthermore, because seizures in BECTS are often brief, and typically nocturnal, accurate clinical reporting of seizures can be a challenge in this population. A reliable physiological biomarker could provide an improved assessment of ongoing seizure risk. In addition, identification of a reliable biomarker of seizure risk enables insight into the pathophysiology of the disease as well as a rapid assay to quantify changes in seizure risk in individual treatment trials and large‐scale clinical trials. As our study was limited by a small sample size, additional studies with larger samples are required to validate our observations. Future longitudinal studies are required to evaluate whether serial changes in beta power may provide an early indication of disease remission.

Consistent with the observation that BECTS is a sleep‐activated disease, we found abnormal cortical rhythms were prominent during sleep in BECTS subjects, but less evident during wakefulness. Among BECTS subjects, we found that beta power decreased during both wakefulness and sleep as these subjects entered remission. These results suggest that altered beta activity may also be present during wakefulness, but fail to distinguish healthy controls from BECTS subjects, potentially due to the increase in noise caused by muscle artifact or other brain activity during wakefulness. As there is high variability in beta power among healthy controls, a larger study would have more power to identify a smaller effect, if present, in the wake data, that remained undetected here. Alternatively, the altered cortical rhythms may be activated by the same processes that support epileptiform activity during sleep (Sanchez‐Vives & McCormick, 2000; Steriade, Contreras, & Amzica, 1994) or the abnormal cortical rhythms themselves may support ictal processes. NREM sleep increases seizure susceptibility in various forms of epilepsy (Bazil & Walczak, 1997; Minecan, Natarajan, Marzec, & Malow, 2002; Shouse, Scordato, & Farber, 2004), though the prominent feature of this sleep state is slower delta and theta range activity (Iber, Ancoli‐Israel, Chesson, & Quan, 2007). Future work to evaluate for subtle differences in beta power during sleep in other sleep activated epilepsy syndromes will clarify the generalizability of this finding.

Our post‐hoc analyses suggest that the abnormalities in cortical rhythms were not restricted to the beta band. Rather, we observed, across large regions of cortex, possible relationships between power in the alpha and theta frequencies in children with BECTS compared to healthy controls. We further observed possible relationships between duration seizure‐free and power in the alpha and gamma frequencies. While GABA‐mediated changes would be expected to involve the beta and gamma bands (Whittington, Traub, Kopell, Ermentrout, & Buhl, 2000), the decrease in power is unlikely to directly reflect changes in GABA activity alone (Baumgarten et al., 2016; Muthukumaraswamy et al., 2013) and the underlying mechanisms supporting these observations remain unknown. We did not find a relationship between beta power and the presence of spikes or spike rate in the seizure onset zone. This observation suggests no strong relationship between these two phenomena; rather epileptiform spikes and beta rhythm abnormalities may reflect independent processes in this disease. Here, we removed all 1 s intervals surrounding spikes from the data to avoid the filter artifact created by sudden, large changes in voltage, as seen with spikes (Kramer et al., 2008). Thus, brief, discrete increases in beta power immediately surrounding spike events could not be ruled out.

Contrary to the expectation that abnormalities in cortical physiology would be restricted to the seizure onset zone in focal epilepsy, we found abnormalities in beta activity across parietal and temporal lobes in children with BECTS compared to healthy controls. Global and frontal lobe beta power decreased with the duration seizure‐free. These findings suggest that the abnormalities in cortical rhythms observed are not discretely tied to the focal epileptiform spikes characteristic of this disease. Rather, consistent with recent observations that BECTS is a complex neuropsychiatric disease involving broad neurocognitive dysfunctions beyond the observed clinical seizures (Wickens et al., 2017), we observe that BECTS rhythm abnormalities may be diffuse, involving multiple cortical regions. We note that we did not find a direct relationship between full scale IQ and global beta power or sensorimotor performance and peri‐rolandic beta power. This finding suggests a strong direct relationship between diffuse cortical rhythms and function does not exist, however in these exploratory analyses there was insufficient power to identify a subtle relationship. As these post‐hoc findings were exploratory, they require validation in a future study.

Finally, we found that electrical source imaging techniques were more sensitive to abnormalities in power compared to sensor space EEG recordings. This is likely due to the spatial blurring of electrical activity during volume conduction from the cortex, through the skull, and to the scalp (Nunez & Srinivasan, 2006). The electrical source imaging algorithm we used, MNE, has been demonstrated to be an accurate and reliable method for localizing sources of EEG activity (Hauk, 2004; Komssi, Huttunen, Aronen, & Ilmoniemi, 2004). On supplementary analysis, we did observe a trend in sensor space analysis, in which beta power tended to decrease with duration seizure‐free. Future work evaluating larger datasets or longitudinal data may provide sufficient sensitivity to detect within subject changes to utilize sensor space recordings for this measure.

## 6 CONCLUSION

Our finding of abnormalities in beta power in the seizure onset zone in children with BECTS compared to healthy controls may provide a novel, accessible, and easily obtainable biomarker to evaluate these patients. Although we found evidence of involvement of broad brain regions and frequency ranges, differences were most consistent in the beta band and in the seizure onset zone. Furthermore, this work supports the utility of quantitative EEG analysis and electrical source imaging techniques to reveal subtle differences in cortical physiology that cannot be appreciated through visual analysis or sensor‐based analysis alone. Future work to evaluate the relationship of cortical rhythms to the underlying disease process in epilepsy will help us better understand whether these findings reflect pathologic or compensatory mechanisms.

## CONFLICTS OF INTEREST

The authors have no conflicts of interest to declare.

## References

[brb31237-bib-0001] Baho, E. , & Di Cristo, G. (2012). Neural activity and neurotransmission regulate the maturation of the innervation field of cortical GABAergic interneurons in an age‐dependent manner. The Journal of Neuroscience, 32(3), 911–918. 10.1523/JNEUROSCI.4352-11.2012 22262889 PMC6621145

[brb31237-bib-0002] Baumgarten, T. J. , Oeltzschner, G. , Hoogenboom, N. , Wittsack, H.‐J. , Schnitzler, A. , & Lange, J. (2016). Beta peak frequencies at rest correlate with endogenous GABA+/Cr concentrations in sensorimotor cortex areas. PLoS ONE, 11(6), e0156829 10.1371/journal.pone.0156829 27258089 PMC4892568

[brb31237-bib-0003] Bazil, C. W. , & Walczak, T. S. (1997). Effects of sleep and sleep stage on epileptic and nonepileptic seizures. Epilepsia, 38(1), 56–62. 10.1111/j.1528-1157.1997.tb01077.x 9024184

[brb31237-bib-0004] Berg, A. T. , Lin, J. , Ebrahimi, N. , Testa, F. M. , Levy, S. R. , & Shinnar, S. (2004). Modeling remission and relapse in pediatric epilepsy: Application of a Markov process. Epilepsy Research, 60(1), 31–40. 10.1016/j.eplepsyres.2004.05.002 15279868

[brb31237-bib-0005] Berg, A. T. , Shinnar, S. , Levy, S. R. , Testa, F. M. , Smith‐Rapaport, S. , Beckerman, B. , & Ebrahimi, N. (2001). Two‐year remission and subsequent relapse in children with newly diagnosed epilepsy. Epilepsia, 42(12), 1553–1562. 10.1046/j.1528-1157.2001.21101.x 11879366

[brb31237-bib-0006] Boor, R. , Jacobs, J. , Hinzmann, A. , Bauermann, T. , Scherg, M. , Boor, S. , … Stoeter, P. (2007). Combined spike‐related functional MRI and multiple source analysis in the non‐invasive spike localization of benign rolandic epilepsy. Clinical Neurophysiology, 118(4), 901–909. 10.1016/j.clinph.2006.11.272 17317297

[brb31237-bib-0007] Bouma, P. A. D. , Bovenkerk, A. C. , Westendorp, R. G. J. , & Brouwer, O. F. (1997). The course of benign partial epilepsy of childhood with centrotemporal spikes: A meta‐analysis. Neurology, 48(2), 430–437. 10.1212/WNL.48.2.430 9040734

[brb31237-bib-0008] Brindley, L. M. , Koelewijn, L. , Kirby, A. , Williams, N. , Thomas, M. , te Water‐Naudé, J. , … Hamandi, K. (2016). Ipsilateral cortical motor desynchronisation is reduced in benign epilepsy with centro‐temporal spikes. Clinical Neurophysiology, 127(2), 1147–1156. 10.1016/j.clinph.2015.08.020 26522940

[brb31237-bib-0009] Chu, C. J. , Kramer, M. A. , Pathmanathan, J. , Bianchi, M. T. , Westover, M. B. , Wizon, L. , & Cash, S. S. (2012). Emergence of stable functional networks in long‐term human electroencephalography. The Journal of Neuroscience, 32(8), 2703–2713. 10.1523/JNEUROSCI.5669-11.2012 22357854 PMC3361717

[brb31237-bib-0010] Chu, C. J. , Leahy, J. , Pathmanathan, J. , Kramer, M. A. , & Cash, S. S. (2014). The maturation of cortical sleep rhythms and networks over early development. Clinical Neurophysiology, 125(7), 1360–1370. 10.1016/j.clinph.2013.11.028 24418219 PMC4035415

[brb31237-bib-0011] Chu, C. J. , Tanaka, N. , Diaz, J. , Edlow, B. L. , Wu, O. , Hämäläinen, M. , … Kramer, M. A. (2015). EEG functional connectivity is partially predicted by underlying white matter connectivity. NeuroImage, 108, 23–33. 10.1016/j.neuroimage.2014.12.033. 25534110 PMC4323839

[brb31237-bib-0012] Ciumas, C. , Saignavongs, M. , Ilski, F. , Herbillon, V. , Laurent, A. , Lothe, A. , … Ryvlin, P. (2014). White matter development in children with benign childhood epilepsy with centro‐temporal spikes. Brain, 137(4), 1095–1106. 10.1093/brain/awu039 24598359

[brb31237-bib-0013] Commission on Classification and Terminology of the International League Against Epilepsy . (1989). Proposal for revised classification of epilepsies and epileptic syndromes. Epilepsia, 30(4), 389–399. 10.1111/j.1528-1157.1989.tb05316.x 2502382

[brb31237-bib-0014] Desikan, R. S. , Ségonne, F. , Fischl, B. , Quinn, B. T. , Dickerson, B. C. , Blacker, D. , … Killiany, R. J. (2006). An automated labeling system for subdividing the human cerebral cortex on MRI scans into gyral based regions of interest. NeuroImage, 31(3), 968–980. 10.1016/j.neuroimage.2006.01.021 16530430

[brb31237-bib-0015] Doumlele, K. , Friedman, D. , Buchhalter, J. , Donner, E. J. , Louik, J. , & Devinsky, O. (2017). Sudden unexpected death in epilepsy among patients with benign childhood epilepsy with centrotemporal spikes. JAMA Neurology, 74(6), 645–649. 10.1001/jamaneurol.2016.6126 28384699 PMC5822211

[brb31237-bib-0016] Engel, A. K. , & Fries, P. (2010). Beta‐band oscillations–signalling the status quo? Current Opinion in Neurobiology, 20(2), 156–165. 10.1016/j.conb.2010.02.015 20359884

[brb31237-bib-0017] Fischl, B. (2012). FreeSurfer. NeuroImage, 62(2), 774–781. 10.1016/j.neuroimage.2012.01.021 22248573 PMC3685476

[brb31237-bib-0018] Forss, N. , Hari, R. , Salmelin, R. , Ahonen, A. , Hämäläinen, M. , Kajola, M. , … Simola, J. (1994). Activation of the human posterior parietal cortex by median nerve stimulation. Experimental Brain Research, 99(2), 309–315. 10.1007/BF00239597 7925811

[brb31237-bib-0019] Freeman, W. J. , Rogers, L. J. , Holmes, M. D. , & Silbergeld, D. L. (2000). Spatial spectral analysis of human electrocorticograms including the alpha and gamma bands. Journal of Neuroscience Methods, 95(2), 111–121. 10.1016/S0165-0270(99)00160-0 10752481

[brb31237-bib-0020] Gasser, T. , Schuller, J. C. , & Gasser, U. S. (2005). Correction of muscle artefacts in the EEG power spectrum. Clinical Neurophysiology, 116(9), 2044–2050. 10.1016/j.clinph.2005.06.002 16043401

[brb31237-bib-0021] Gramfort, A. , Luessi, M. , Larson, E. , Engemann, D. A. , Strohmeier, D. , Brodbeck, C. , … Hämäläinen, M. S. (2014). MNE software for processing MEG and EEG data. NeuroImage, 86, 446–460. 10.1016/J.NEUROIMAGE.2013.10.027 24161808 PMC3930851

[brb31237-bib-0022] Halma, E. , De Louw, A. J. A. , Klinkenberg, S. , Aldenkamp, A. P. , Ijff, D. M. , & Majoie, M. (2014). Behavioral side‐effects of levetiracetam in children with epilepsy: A systematic review. Seizure, 23(9), 685–691. 10.1016/j.seizure.2014.06.004 24981629

[brb31237-bib-0023] Hamalainen, M. S. , & Sarvas, J. (1989). Realistic conductivity geometry model of the human head for interpretation of neuromagnetic data. IEEE Transactions on Biomedical Engineering, 36(2), 165–171. 10.1109/10.16463 2917762

[brb31237-bib-0024] Hauk, O. (2004). Keep it simple: A case for using classical minimum norm estimation in the analysis of EEG and MEG data. NeuroImage, 21(4), 1612–1621. 10.1016/J.NEUROIMAGE.2003.12.018 15050585

[brb31237-bib-0025] He, B. J. , Zempel, J. M. , Snyder, A. Z. , & Raichle, M. E. (2010). The temporal structures and functional significance of scale‐free brain activity. Neuron, 66(3), 353–369. 10.1016/J.NEURON.2010.04.020 20471349 PMC2878725

[brb31237-bib-0026] Iber, C. , Ancoli-Israel, S. , Chesson, A. L. , & Quan, S. F. (2007). The AASM manual for the scoring of sleep and associated events: Rules, terminology, and technical specifications. Westchester, NY: American Academy of Sleep Medicine.

[brb31237-bib-0027] Jensen, O. , Goel, P. , Kopell, N. , Pohja, M. , Hari, R. , & Ermentrout, B. (2005). On the human sensorimotor‐cortex beta rhythm: Sources and modeling. NeuroImage, 26(2), 347–355. 10.1016/j.neuroimage.2005.02.008 15907295

[brb31237-bib-0028] Khazipov, R. , Khalilov, I. , Tyzio, R. , Morozova, E. , Ben‐Ari, Y. , & Holmes, G. L. (2004). Developmental changes in GABAergic actions and seizure susceptibility in the rat hippocampus. European Journal of Neuroscience, 19(3), 590–600. 10.1111/j.0953-816X.2003.03152.x 14984409

[brb31237-bib-0029] Kim, E.‐H. , Yum, M.‐S. , Shim, W.‐H. , Yoon, H.‐K. , Lee, Y.‐J. , & Ko, T.‐S. (2015). Structural abnormalities in benign childhood epilepsy with centrotemporal spikes (BCECTS). Seizure, 27, 40–46. 10.1016/j.seizure.2015.02.027 25891925

[brb31237-bib-0030] Kim, H. , Kim, S. Y. , Lim, B. C. , Hwang, H. , Chae, J. H. , Choi, J. , … Dlugos, D. J. (2018). Spike persistence and normalization in benign epilepsy with centrotemporal spikes – implications for management. Brain Dev, 40(8), 693–698. 10.1016/j.braindev.2018.04.011 29754875

[brb31237-bib-0031] Kim, S. E. , Lee, J. H. , Chung, H. K. , Lim, S. M. , & Lee, H. W. (2014). Alterations in white matter microstructures and cognitive dysfunctions in benign childhood epilepsy with centrotemporal spikes. European Journal of Neurology, 21(5), 708–717. 10.1111/ene.12301 24330132

[brb31237-bib-0032] Kobayashi, K. , Yoshinaga, H. , Toda, Y. , Inoue, T. , Oka, M. , & Ohtsuka, Y. (2010). High-frequency oscillations in idiopathic partial epilepsy of childhood. Epilepsia, 52, 1812–1819. 10.1111/j.1528-1167.2011.03169.x 21762448

[brb31237-bib-0033] Koelewijn, L. , Hamandi, K. , Brindley, L. M. , Brookes, M. J. , Routley, B. C. , Muthukumaraswamy, S. D. , … Singh, K. D. (2015). Resting‐state oscillatory dynamics in sensorimotor cortex in benign epilepsy with centro‐temporal spikes and typical brain development. Human Brain Mapping, 36(10), 3935–3949. 10.1002/hbm.22888 26177579 PMC6869151

[brb31237-bib-0034] Komssi, S. , Huttunen, J. , Aronen, H. J. , & Ilmoniemi, R. J. (2004). EEG minimum‐norm estimation compared with MEG dipole fitting in the localization of somatosensory sources at S1. Clinical Neurophysiology, 115(3), 534–542. 10.1016/j.clinph.2003.10.034 15036048

[brb31237-bib-0035] Kramer, M. A. , & Eden, U. T. (2016). Case studies in neural data analysis: A guide for the practicing neuroscientist. Cambridge, MA: MIT Press.

[brb31237-bib-0036] Kramer, M. A. , Tort, A. B. L. , & Kopell, N. J. (2008). Sharp edge artifacts and spurious coupling in EEG frequency comodulation measures. Journal of Neuroscience Methods, 170, 352–357. 10.1016/j.jneumeth.2008.01.020 18328571

[brb31237-bib-0037] LaPlante, R. A. , Tang, W. , Peled, N. , Vallejo, D. , Borzello, M. , Dougherty, D. D. , … Stufflebeam, S. M. (2017). The interactive electrode localization utility: software for automatic sorting and labeling of intracranial subdural electrodes. International Journal of Computer Assisted Radiology and Surgery, 12, 1829–1837. 10.1007/s11548-016-1504-2 27915398 PMC5777932

[brb31237-bib-0038] Le Magueresse, C. , & Monyer, H. (2013). GABAergic interneurons shape the functional maturation of the cortex. Neuron, 77(3), 388–405. 10.1016/J.NEURON.2013.01.011 23395369

[brb31237-bib-0039] Lepage, K. Q. , Kramer, M. A. , & Chu, C. J. (2014). A statistically robust EEG re‐referencing procedure to mitigate reference effect. Journal of Neuroscience Methods, 235, 101–116. 10.1016/j.jneumeth.2014.05.008 24975291 PMC4160811

[brb31237-bib-0040] Lin, Y. , Shih, Y. , Chang, K. , Lee, W. , Yu, H. , Hsieh, J. , … Ho, L. (2003). MEG localization of rolandic spikes with respect to SI and SII cortices in benign rolandic epilepsy. NeuroImage, 20(4), 2051–2061. 10.1016/J.NEUROIMAGE.2003.08.019 14683709

[brb31237-bib-0041] Masur, D. , Shinnar, S. , Cnaan, A. , Shinnar, R. c. , Clark, P. , Wang, J. , … Childhood Absence Epilepsy Study Group . (2013). Pretreatment cognitive deficits and treatment effects on attention in childhood absence epilepsy. Neurology, 81(18), 1572–1580. 10.1212/WNL.0b013e3182a9f3ca 24089388 PMC3806916

[brb31237-bib-0042] Minecan, D. , Natarajan, A. , Marzec, M. , & Malow, B. (2002). Relationship of epileptic seizures to sleep stage and sleep depth. Sleep, 25(8), 56–61. 10.1093/sleep/25.8.56 12489898

[brb31237-bib-0043] Muthukumaraswamy, S. D. , Myers, J. F. M. , Wilson, S. J. , Nutt, D. J. , Lingford‐Hughes, A. , Singh, K. D. , & Hamandi, K. (2013). The effects of elevated endogenous GABA levels on movement‐related network oscillations. NeuroImage, 66, 36–41. 10.1016/j.neuroimage.2012.10.054 23110884

[brb31237-bib-0044] Nunez, P. L. , & Srinivasan, R. (2006). Electric fields of the brain: The neurophysics of EEG. Electric fields of the brain: The neurophysics of EEG. New York: Oxford University Press Inc.

[brb31237-bib-0045] Overvliet, G. M. , Besseling, R. M. H. , Jansen, J. F. A. , Van Der Kruijs, S. J. M. , Vles, J. S. H. , Hofman, P. A. M. , … Backes, W. H. (2013). Early onset of cortical thinning in children with rolandic epilepsy. NeuroImage: Clinical, 2(1), 434–439. 10.1016/j.nicl.2013.03.008 24179797 PMC3777705

[brb31237-bib-0046] Panayiotopoulos, C. P. , Michael, M. , Sanders, S. , Valeta, T. , & Koutroumanidis, M. (2008). Benign childhood focal epilepsies: Assessment of established and newly recognized syndromes. Brain, 131(9), 2264–2286. 10.1093/brain/awn162 18718967

[brb31237-bib-0047] Pardoe, H. R. , Berg, A. T. , Archer, J. S. , Fulbright, R. K. , & Jackson, G. D. (2013). A neurodevelopnnental basis for BECTS: Evidence from structural MRI. Epilepsy Research, 105(1–2), 133–139. 10.1016/j.eplepsyres.2012.11.008 23375559 PMC3669634

[brb31237-bib-0048] Pataraia, E. , Feucht, M. , Lindinger, G. , Aull‐Watschinger, S. , & Baumgartner, C. (2008). Combined electroencephalography and magnetoencephalography of interictal spikes in benign rolandic epilepsy of childhood. Clinical Neurophysiology, 119(3), 635–641. 18164663 10.1016/j.clinph.2007.11.009

[brb31237-bib-0049] Perry, S. , Holt, P. , & Benatar, M. (2008). Levetiracetam versus carbamazepine monotherapy for partial epilepsy in children less than 16 years of age. Journal of Child Neurology, 23(5), 515–519. 10.1177/0883073807309784 18182645

[brb31237-bib-0050] Sanchez‐Vives, M. V. , & McCormick, D. A. (2000). Cellular and network mechanisms of rhythmic recurrent activity in neocortex. Nature Neuroscience, 3(10), 1027–1034. 10.1038/79848 11017176

[brb31237-bib-0051] Scheffer, I. E. , Berkovic, S. , Capovilla, G. , Connolly, M. B. , French, J. , Guilhoto, L. , … Zuberi, S. M. (2017). ILAE classification of the epilepsies: Position paper of the ILAE Commission for Classification and Terminology. Epilepsia, 58(4), 512–521. 10.1111/epi.13709 28276062 PMC5386840

[brb31237-bib-0052] Sharon, D. , Hämäläinen, M. S. , Tootell, R. B. H. , Halgren, E. , & Belliveau, J. W. (2007). The advantage of combining MEG and EEG: Comparison to fMRI in focally stimulated visual cortex. NeuroImage, 36(4), 1225–1235. 10.1016/J.NEUROIMAGE.2007.03.066 17532230 PMC2706118

[brb31237-bib-0053] Shields, D. W. , & Carter Snead III, O. (2009). Benign epilepsy with centrotemporal spikes. Epilepsia, 50(s8), 10–15. 10.1111/j.1528-1167.2009.02229.x 19702727

[brb31237-bib-0054] Shiraishi, H. , Haginoya, K. , Nakagawa, E. , Saitoh, S. , Kaneko, Y. , Nakasato, N. , … Otsubo, H. (2014). Magnetoencephalography localizing spike sources of atypical benign partial epilepsy. Brain & Development, 36(1), 21–27. 10.1016/j.braindev.2012.12.011 23384398

[brb31237-bib-0055] Shouse, M. N. , Scordato, J. C. , & Farber, P. R. (2004). Sleep and arousal mechanisms in experimental epilepsy: Epileptic components of NREM and antiepileptic components of REM sleep. Mental Retardation and Developmental Disabilities Research Reviews, 10(2), 117–121. 10.1002/mrdd.20022 15362167

[brb31237-bib-0056] Silber, M. H. , Ancoli‐Israel, S. , Bonnet, M. H. , Chokroverty, S. , Grigg‐Damberger, M. M. , Hirshkowitz, M. , … Iber, C. (2007). The visual scoring of sleep in adults. Journal of Clinical Sleep Medicine. 3, 22. 17557422

[brb31237-bib-0057] Steriade, M. , Contreras, D. , & Amzica, F. (1994). Synchronized sleep oscillations and their paroxysmal developments. Trends in Neurosciences, 17(5), 201–207. 10.1016/0166-2236(94)90105-8 7520202

[brb31237-bib-0058] Whittington, M. A. , Traub, R. D. , Kopell, N. , Ermentrout, B. , & Buhl, E. H. (2000). Inhibition‐based rhythms: Experimental and mathematical observations on network dynamics. International Journal of Psychophysiology, 38, 315–336. 10.1016/S0167-8760(00)00173-2 11102670

[brb31237-bib-0059] Wickens, S. , Bowden, S. C. , & D'Souza, W. (2017). Cognitive functioning in children with self‐limited epilepsy with centrotemporal spikes: A systematic review and meta‐analysis. Epilepsia, 58(10), 1673–1685. 10.1111/epi.13865 28801973

[brb31237-bib-0060] Xiao, F. , Chen, Q. , Yu, X. , Tang, Y. , Luo, C. , Fang, J. , … Zhou, D. (2014). Hemispheric lateralization of microstructural white matter abnormalities in children with active benign childhood epilepsy with centrotemporal spikes (BECTS): A preliminary DTI study. Journal of the Neurological Sciences, 336, 171–179. 10.1016/j.jns.2013.10.033 24210075

[brb31237-bib-0061] Xie, W. , Ross, E. E. , Kramer, M. A. , Eden, U. T. , & Chu, C. J. (2018). Iming matters: Impact of anticonvulsant drug treatment and spikes on seizure risk in benign epilepsy with centrotemporal spikes. Epilepsia Open, 3, 409–417. 30187012 10.1002/epi4.12248 PMC6119752

[brb31237-bib-0062] Yao, J. , & Dewald, J. P. A. (2005). Evaluation of different cortical source localization methods using simulated and experimental EEG data. NeuroImage, 25(2), 369–382. 10.1016/j.neuroimage.2004.11.036 15784415

