# Supplementary figures and images for: Beta oscillations in the sensorimotor cortex correlate with disease and remission in benign epilepsy with centrotemporal spikes
Source: Brain Behav. 2019 Feb 20;9(3):e01237. doi: 10.1002/brb3.1237 (PMC6422718; doi:10.1002/brb3.1237)

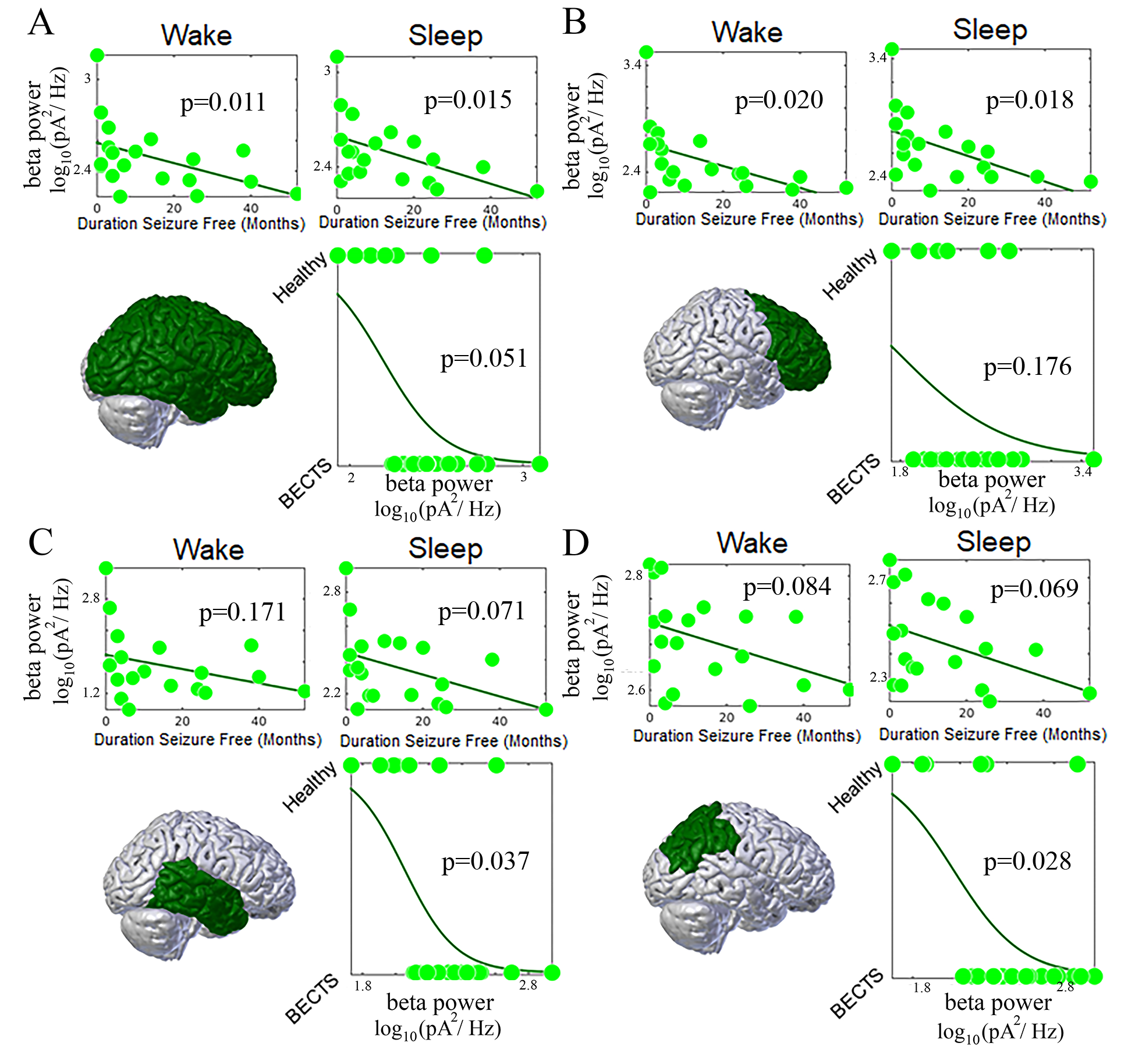

Supplement: Supplementary file 1 [file BRB3-9-e01237-s001.tif]

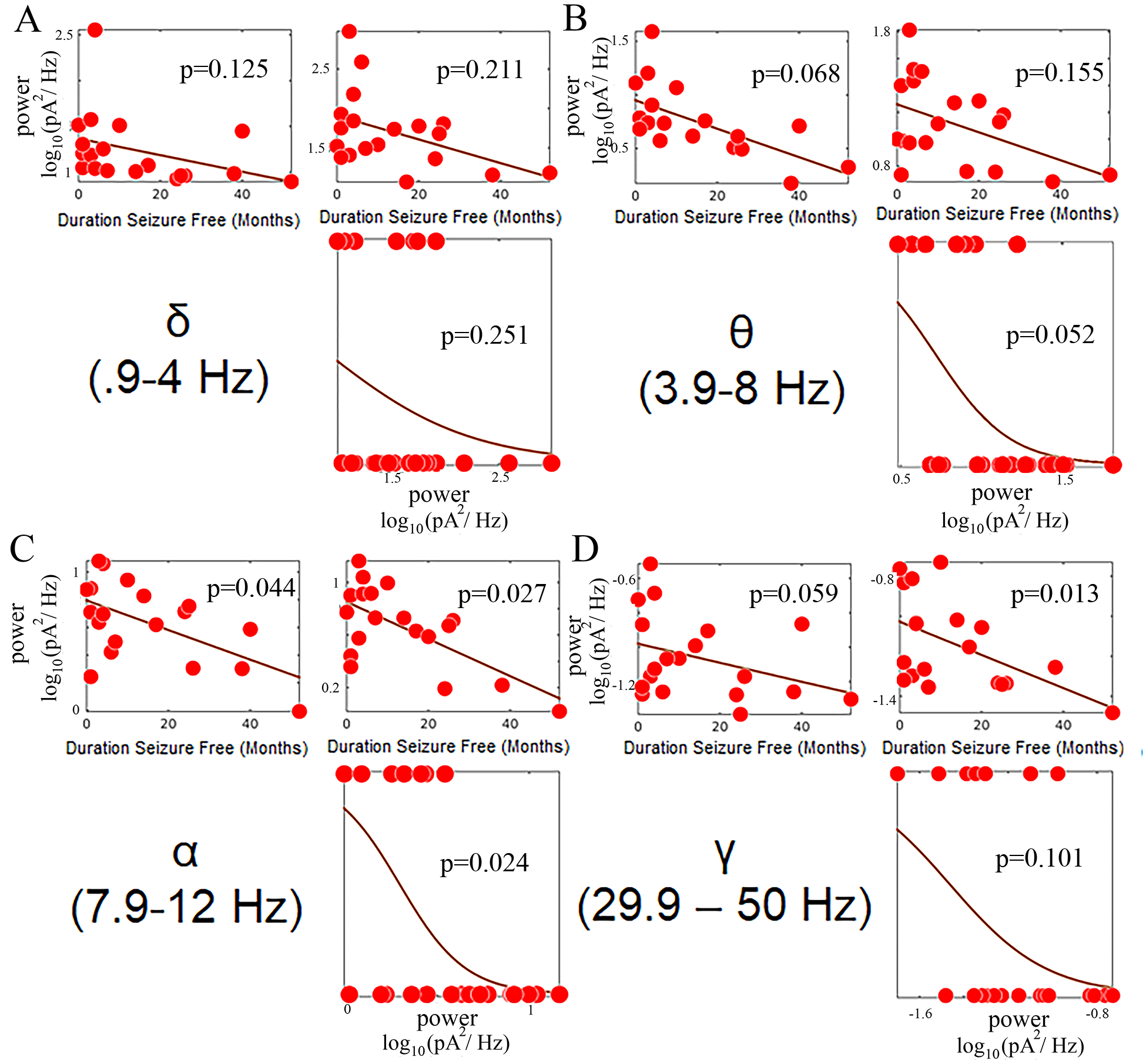

Supplement: Supplementary file 2 [file BRB3-9-e01237-s002.tif]

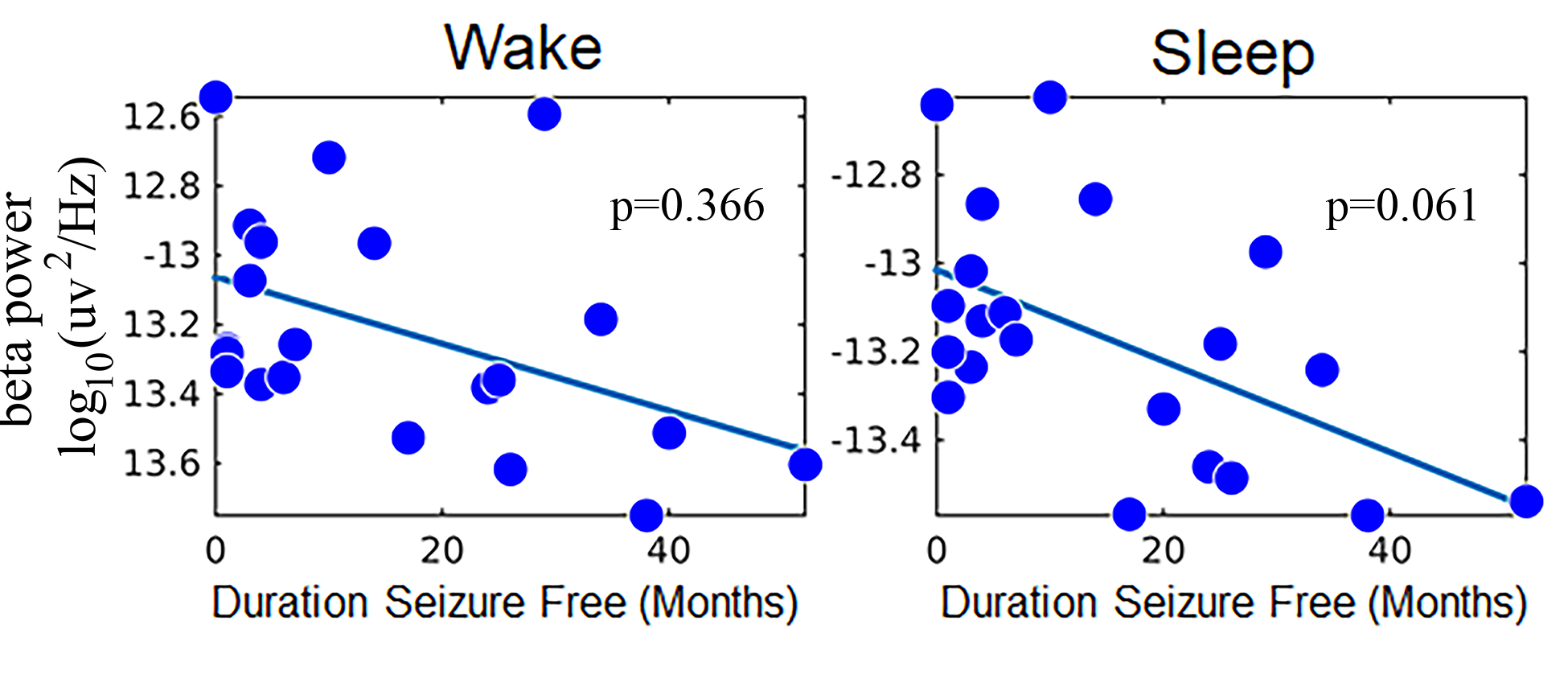

Supplement: Supplementary file 3 [file BRB3-9-e01237-s003.tif]
