# Supplementary material for: Beta oscillations in the sensorimotor cortex correlate with disease and remission in benign epilepsy with centrotemporal spikes
Source: Brain Behav. 2019 Feb 20;9(3):e01237. doi: 10.1002/brb3.1237 (PMC6422718; doi:10.1002/brb3.1237)
Supplement: Supplementary file 4 [file BRB3-9-e01237-s004.docx]

|  |  |  |  |  |  |
| --- | --- | --- | --- | --- | --- |
| **Spatial Specificity** | Dependent variable | Lobe | Univariate  Beta power | Univariate  Age | Multivariate  Beta power  (Age as covariate) |
| Logistic regression | Healthy Control or BECTS | Global | **p=0.051^**  (sleep) | p=0.846 |  |
|  |  | Frontal | p=0.176  (sleep) |  |  |
|  |  | Temporal | **p=0.037^**  (sleep) |  |  |
|  |  | Parietal | **p=0.028^**  (sleep) |  |  |
|  |  | Occipital | p=0.189  (sleep) |  |  |
| Linear regression | Duration Seizure-Free | Global | p=0.043  (wake) | p=0.003 | **p=.011^** |
|  |  |  | p=0.026  (sleep) | p=0.011 | **p=.015^** |
|  |  | Frontal | p=0.025  (wake) |  | **p=.020^** |
|  |  |  | p=0.018  (sleep) |  | **p=.018^** |
|  |  | Parietal | p=0.084  (wake) |  |  |
|  |  |  | p=0.069  (sleep) |  |  |
|  |  | Temporal | p=0.171  (wake) |  |  |
|  |  |  | p=0.071  (sleep) |  |  |
|  |  | Occipital | p=0.763  (wake) |  |  |
|  |  |  | p=0.800  (sleep) |  |  |

Supplementary Table 1. Post-hoc analysis to explore spatial distribution of beta power abnormalities. We note that the same subjects were tested for all analyses, so age was tested once for the logistic and linear models, respectively. We also note that these tests were exploratory and not corrected for multiple comparisons. ^ indicates a possible relationship.

|  |  |  |  |  |  |
| --- | --- | --- | --- | --- | --- |
| **Frequency Specificity** | Dependent variable | Frequency band | Univariate  Beta power | Univariate  Age | Multivariate  Beta power  (Age as covariate) |
| Logistic regression | Healthy Control or BECTS | Delta | p=0.251  (sleep) | p=0.846 |  |
|  |  | Theta | p=0.052  (sleep) |  |  |
|  |  | Alpha | **p=0.024^ (sleep)** |  |  |
|  |  | Gamma | p=0.101  (sleep) |  |  |
| Linear regression | Duration Seizure-Free | Delta | p=0.125  (wake) | p=0.003 |  |
|  |  |  | p=0.047  (sleep) | p=0.011 | Freq p=0.211 |
|  |  | Theta | p=0.003  (wake) |  | Freq p=0.068 |
|  |  |  | p=0.042  (sleep) |  | Freq p=0.155 |
|  |  | Alpha | p=0.010  (wake) |  | **Freq p=0.044^** |
|  |  |  | p=0.002  (sleep) |  | **Freq p=0.027^** |
|  |  | Gamma | p=0.136  (wake) |  | Freq p=0.059 |
|  |  |  | p=0.021  (sleep) |  | **Freq p=0.013^** |

Supplementary Table 2. Post-hoc analysis to explore rhythm abnormalities across frequencies. We note that the same subjects were tested for all analyses, so age was tested once for the logistic and linear models, respectively. We also note that these tests were exploratory and not corrected for multiple comparisons. ^ indicates a possible relationship.

|  |  |  |  |  |
| --- | --- | --- | --- | --- |
| **Sensor space** | Dependent variable | Univariate  Beta power | Univariate  Age | Multivariate  Beta power  (Age as covariate) |
| Logistic regression | Healthy control or BECTS  (central channels) | p=0.523  (beta, wake) | p=0.173 |  |
|  |  | p=0.264  (beta, sleep) | p=0.983 |  |
| Linear regression | Duration Seizure-Free  (central channels) | p=0.040  (wake)  p=0.011 | p=0.017 | beta p=0.336  beta p=0.061 |
| Linear regression | Duration Seizure-Free  (channel subset from all lobes) | p=0.339  (wake) | p=0.017 | beta p=0.965 |
|  |  | p=0.074  (sleep) | p=0.047 | beta p=0.201 |
| Linear regression | Duration Seizure-Free  (central channels) | p=0.065  (delta, sleep)  p=0.064  (theta, sleep)  p=0.027  (alpha, sleep) | p=0.047 | alpha p=0.142 |
|  |  | p=0.038 |  | gamma p=0.081 |
|  |  | (gamma, sleep) |  |  |

Supplementary Table 3. Post-hoc analysis to explore for rhythm abnormalities in sensor space across frequencies and all channels. We note that the same subjects were tested for all analyses, so age was tested once for the logistic and linear models, respectively.

|  |  |  |  |  |
| --- | --- | --- | --- | --- |
| **Clinical variables** | Dependent variable | Univariate  Beta power | Univariate  Age | Multivariate  Beta power  (Age as covariate) |
| Logistic regression | Presence of spikes | p=0.878 |  |  |
|  |  |  |  |  |
| Linear regression | Spike rate | p=0.053 | p=0.011 | p=0.203 |
|  |  |  |  |  |
| Logistic regression | Neuropsychological diagnosis  Grooved Peg Board performance (dominant hand) | p=0.674 |  |  |
| Linear regression |  | P=0.540 |  |  |
|  |  |  |  |  |

Supplementary Table 4. Post-hoc analysis to explore for beta abnormalities in the seizure onset zone and clinical features.
